# Relationships between Heavy Metal Concentrations in Roadside Topsoil and Distance to Road Edge Based on Field Observations in the Qinghai-Tibet Plateau, China

**DOI:** 10.3390/ijerph10030762

**Published:** 2013-02-25

**Authors:** Xuedong Yan, Dan Gao, Fan Zhang, Chen Zeng, Wang Xiang, Man Zhang

**Affiliations:** 1 MOE Key Laboratory for Urban Transportation Complex Systems Theory and Technology, Beijing Jiaotong University, Beijing 100044, China; E-Mails: 11121095@bjtu.edu.cn (D.G.); 12114242@bjtu.edu.cn (W.X.); 10125479@bjtu.edu.cn (M.Z.); 2 Key Laboratory of Tibetan Environment Changes and Land Surface Processes, Institute of Tibetan Plateau Research, Chinese Academy of Sciences, Beijing 100101, China; E-Mails: zhangfan@itpcas.ac.cn (F.Z.); zengchen@itpcas.ac.cn (C.Z.)

**Keywords:** Qinghai-Tibet Plateau, heavy metal, traffic emissions, exponential model, potential ecological risk

## Abstract

This study investigated the spatial distribution of copper (Cu), zinc (Zn), cadmium (Cd), lead (Pb), chromium (Cr), cobalt (Co), nickel (Ni) and arsenic (As) in roadside topsoil in the Qinghai-Tibet Plateau and evaluated the potential environmental risks of these roadside heavy metals due to traffic emissions. A total of 120 topsoil samples were collected along five road segments in the Qinghai-Tibet Plateau. The nonlinear regression method was used to formulize the relationship between the metal concentrations in roadside soils and roadside distance. The Hakanson potential ecological risk index method was applied to assess the degrees of heavy metal contaminations. The regression results showed that both of the heavy metals’ concentrations and their ecological risk indices decreased exponentially with the increase of roadside distance. The large R square values of the regression models indicate that the exponential regression method can suitably describe the relationship between heavy metal accumulation and roadside distance. For the entire study region, there was a moderate level of potential ecological risk within a 10 m roadside distance. However, Cd was the only prominent heavy metal which posed potential hazard to the local soil ecosystem. Overall, the rank of risk contribution to the local environments among the eight heavy metals was Cd > As > Ni > Pb > Cu > Co > Zn > Cr. Considering that Cd is a more hazardous heavy metal than other elements for public health, the local government should pay special attention to this traffic-related environmental issue.

## 1. Introduction

Heavy metals are typical road traffic source contaminants in the local ecological environments and thus threaten public heath [1]. These metals are found in fuels, fuel tanks, engines and other vehicle components, catalytic converters, tires and brake pads, as well as in road surface materials [2]. Heavy-metal contaminants can easily impact people residing within the vicinity of the roads via suspended dust or direct contract [3]. If there are farmlands within the scope that the contaminants can reach, they may enter the food chain as a result of their uptake by edible plants [4], thus causing serious health risks. Because of their toxicity (especially for Cd and Pb), persistence and non-degradability characteristics, it is of great importance to monitor the heavy metals concentrations in roadside environments.

Roadside soils are the major reservoirs of traffic-related heavy metals [3]. The concentrations of heavy metals in roadside soils are indicators of heavy metals’ accumulation through atmospheric deposition and road runoff [5]. Generally, the topsoil adjacent to the road edge is collected for analyzing the heavy metals pollution levels. Most observational studies on the concentrations of heavy metals in roadside soils were focused on Cu, Zn, Cd, and Pb [6,7,8,9]. Some research extended the monitored metals to Cr, Ni and As [3,10,11].

Monitoring studies on roadside heavy metal contamination have been conducted in many important cities in China, such as Hong Kong [12,13,14], Beijing [3], and Shanghai [15], as well as the regions in the other countries including Mexico City [16], Turkey’s Elazig [4], England’s Yorkshire [8], Jordan’s Amman [17], Greece’s Kavala [5], *etc*. The major objectives of these studies were to identify the spatial distribution patterns and pollution ranges of heavy metals in roadside soils in order to provide a scientific basis for environmental risk assessment and public health management. Especially in agricultural regions, the assessment and control for potential ecological risks of heavy metals in soils in terms of roadside distance are meaningful for food safety. 

Many previous studies concluded that the heavy metal content in roadside soil has a belt-shaped distribution in terms of distance to road edge, decreasing exponentially with increment of roadside distance [6,7,8,10,11,18,19]. However, other research results showed that the special distribution patterns of heavy metals in roadside soils were not always significantly correlated with the roadside distance [3,4,9]. This may be attributed to mixed sources of metals, agricultural activities and roadside green belts [9]. For the different types of heavy metals, their maximum influential roadside distance may vary substantially. Generally, most of the metals’ influential roadside distances are less than 50 m, but may be up to 100 m [19,20]. Pb may even have an impact on roadside soil up to 320 m away from the road [7]. Therefore, it is suggested that the spatial distribution patterns of heavy metals owing to the traffic source should be investigated in the areas without disturbances of other contamination sources. 

In this study, we choose the Qinghai-Tibet Plateau as the research region to explore the relationship between heavy metal concentrations in soils and roadside distance. The Qinghai-Tibet Plateau and surrounding mountains are named as the Third Pole of the World due to its high altitude (over 4,000 m), large area of more than 5 million km^2^, unique geographical position, and special climate environment effects [21,22]. The change of the Tibetan Plateau environment strongly influences its alpine ecosystem and responds sensitively to global climate change. Because there are few industrial activities and low density population in this region, its environment is least affected by anthropogenic activities. However, with the increasing tourism and frequent goods exchanges with inland areas, road transportation and construction are rapidly developing in the region. Road traffic has become the most important source of heavy metals in roadside soils at the Qinghai-Tibet Plateau. Thus, dynamic monitoring of heavy-metal pollution due to vehicle emissions has becomes more important to its alpine ecosystem. 

In the Qinghai-Tibet Plateau, we selected observation sites without any other anthropogenic confounding pollution sources (e.g., industrial emissions, residential activities, agriculture or stockbreeding) along five major road segments. Since the traffic emission can be considered as a sole pollution source of heavy metals in the roadside topsoil, this investigation can more clearly display spatial relationships between the heavy metals’ accumulation and roadside distance. 

## 2. Materials and Methods

### 2.1. Study Area and Field Sampling

**Figure 1:**
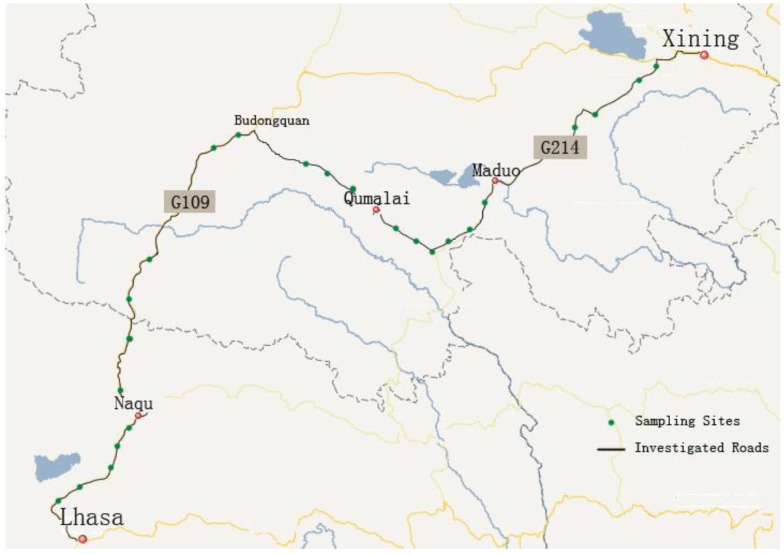
The sampling site distribution in the study area.

The data were collected along five road segments in the Qinghai-Tibet Plateau (31°16'N–31°38'N, 91°34'E–92°21'E, 4,522 m–4,578 m above sea level), China. The road segments are from Xining to Maduo along 214# national highway, from Maduo to Qumalai, from Qumalai to Budongquan of 308# provincial highway, from Budongquan to Naqu of 109# national highway and from Naqu to Lhasa of 109# national highway, as shown in Figure 1. Although the data about daily traffic volume in the road segments were not available, it was clear that the level of traffic volume in the 109# national highway was highest, followed by the 214# national highway, and the level of traffic volume in the 308# provincial highway was the lowest. The 109# national highway, also called the Qinghai-Tibet Highway, is the most important transportation route in the Qinghai-Tibet Plateau, with more than 50 years of history, while the 308# provincial highway was constructed just in recent years. Furthermore, most of the soils along the studied roads are meadow soils, which have sufficient moisture and organic matter.

In order to ensure that the sampling sites are representative, the samples were collected at intervals of 30 kilometers along the routes. At each site, the roadside terrain is flat and widely open within a scope of not less than 200 m. In addition, there is no residential community, farmland, stockbreeding region, mining or other industrial facilities nearby the sampling sites. According to these criteria, a total of 24 sampling sites were identified in the study region, as shown in Figure 1. For each site, five topsoil (0–5 cm) samples were collected at 0 m, 10 m, 30 m, 50 m and 100 m roadside distances, respectively. Each sample was composed of 8–10 sub-samples that were taken in an ‘S-shape’ pattern in a 10 m × 2 m plot and evenly mixed. A total of 120 soil samples were carried back to the laboratory for chemical analyses of heavy metal concentrations in the soils. The metal elements studied in this paper include Cu, Zn, Cd, Pb, Cr, Co, Ni and As. 

### 2.2. Chemical Analysis and Quality Control

The 120 soil samples were dried at room temperature and then ground with an agate mortar until all particles passed a 100 mesh nylon sieve. The 0.3 ± 0.0001 g soil of each ground sample was digested in mixed acid liquid (6 mL HNO_3_–3 mL HCL–0.25 mL H_2_O_2_) in digestion vessels, and decomposed with a full automatic microwave. Finally, the total content of metals was analyzed by Inductively Coupled Plasma-Mass Spectrometry (ICP-MS, Thermo X Series 2). 

Quality assurance and quality control for the chemical analyses were performed according to GSS-8 (Geochemical Standard Reference Sample Soil). Reagent blanks were used as control with each batch of samples assayed in the chemical analyses. In addition, the acid used in chemical analysis was guaranteed reagent grade. To avoid the potential external contamination during the process, all of the vessels used in chemical analysis only containing mixed acid liquid (6 mL HNO_3_–3 mL HCL–0.25 mL H_2_O_2_) were decomposed with a full automatic microwave, the same as the procedure for soil sample decomposition. Subsequently, they were washed with deionized water, then with Milli-Q water for three times. The recovery percentages of heavy metal concentrations from GSS-8 were between 94–105% for Cu, 93–108% for Zn, 93–111% for Cd, 97–104% for Pb, 95–104% for Cr, 93–104% for Co, 92–106% for Ni and 92–108% for As. 25% of the soil samples were analyzed three times to determine the precision of the analytical process. A standard deviation that ranges within 5% was considered satisfactory for environmental analyses.

### 2.3. Data Analysis Methods

#### 2.3.1. The Nonlinear Regression Method

In this study, the nonlinear regression model was used to characterize and predict the relationship between metal concentrations and roadside distance. The Asymptotic Regression was applied to formulize the variation trend of metal concentrations. The formula is shown in Equation (1):

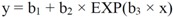
(1)
where y is the metal concentration, x is the roadside distance, and b_1_, b_2_, and b_3_ are the model coefficients. 

#### 2.3.2. The Hakanson Potential Ecological Risk Index Method

The Hakanson potential ecological risk index method was used to assess the contamination degrees of heavy metals for the study regions. The index is calculated by Equation (2):

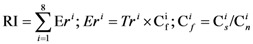
(2)
where RI is the potential ecological risk index for a given region; Er^i^ is the potential ecological risk factor of a given substance; Tr^i^ is the toxic-response factor of a single pollution element (We use the toxicity coefficient of heavy metal [23]); Ci f is the contamination factor of one heavy metal; Ci s is the measured concentration of pollution element in the sediments (mg·kg^−1^); Ci n is the background value of heavy metal in meadow soil in this study. The ecological risk (Er^i^) degree of a given substance was divided into 5 grades and the potential ecological risk (RI) degree of a given region was divided into 4 grades [24], as shown in Table 1. The background values of Cu, Zn, Cd, Pb, Cr, Co, Ni and As are 19.8, 70, 0.084, 22.4, 51.1, 11.8, 23.3 and 8.8, respectively [25]. The toxic coefficients of Cu, Zn, Cd, Pb, Cr, Co, Ni and As are 5, 1, 30, 5, 2, 5, 5 and 10, commonly adopted in the previous studies [23].

**Table 1:** The relationship of Er^i^, RI and pollution degree.

Er^i^	Pollution Degree	RI	Pollution Degree
Er^i^ < 40	low potential ecological risk	RI < 150	low ecological risk
40 ≤ Er^i^ < 80	moderate potential ecological risk	150 ≤ RI < 300	moderate ecological risk
80 ≤ Er^i^ < 160	considerable potential ecological risk	300 ≤ RI < 600	considerable ecological risk
160 ≤ Er^i^ < 320	high potential ecological risk	RI ≥ 600	very high ecological risk
Er^i^ > 320	very high ecological risk		

## 3. Results and Discussion

### 3.1. Concentrations of Soil Heavy Metals on Different Road Segments

The basic statistical descriptions of heavy metals concentrations (mg·kg^−1^) for different road segments in roadside soils are summarized in Table 2. The mean concentrations of Cu (23.13 mg·kg^−1^), Zn (100.97 mg·kg^−1^), Cd (0.30 mg·kg^−1^), Pb (29.60 mg·kg^−1^), Ni (31.93 mg·kg^−1^) and As (21.40 mg·kg^−1^) of the entire region were higher than the background values [25] and the concentrations of Cr (36.20 mg·kg^−1^) and Co (10.31 mg·kg^−1^) were lower than the background values. The concentrations of Zn, Cd and As along the Budongquan-Naqu road segment were the highest among the five road segments, while the concentrations of Pb and Cr along the Naqu-Lhasa road segment were the highest. The finding is in accordance with the levels of traffic volume in these road segments. These road segments from Buqongquan to Naqu to Lhasa are parts of the Qinghai-Tibet Highway. The traffic volume of these two segments should be much higher than the other three segments. 

**Table 2:** Concentrations of roadside soil heavy metals on different road segments.

Road Segment	Site	Concentrations (mg·kg^−1^)							
		Cu	Zn	Cd	Pb	Cr	Co	Ni	As
Xining To Maduo	1	28.33	101.10	0.35	24.58	36.37	10.01	26.66	23.52
	2	28.88	81.57	0.23	18.73	38.84	10.61	29.47	17.35
	3	25.03	85.75	0.26	20.64	36.35	10.14	27.76	16.25
	4	25.59	88.71	0.24	20.85	40.03	10.56	29.58	19.45
Mean		26.96	89.28	0.27	21.20	37.90	10.33	28.37	19.14
Maduo To Qumalai	5	20.43	87.08	0.20	19.07	32.85	10.05	28.67	18.17
	6	21.09	97.30	0.17	15.86	41.46	8.70	31.29	14.37
	7	23.35	93.15	0.28	20.97	37.06	10.51	30.35	23.07
	8	19.11	87.87	0.20	17.88	26.24	8.94	26.43	16.09
	9	26.39	116.83	0.23	25.15	38.10	14.54	37.61	27.74
	10	23.75	96.81	0.22	23.01	35.07	11.78	32.32	23.47
Mean		22.35	96.51	0.22	20.32	35.13	10.75	31.11	20.49
Qumalai To Budongquan	11	23.48	93.40	0.23	22.38	34.49	11.14	35.32	22.97
	12	26.33	106.47	0.27	23.41	31.78	11.06	31.51	23.95
	13	17.41	79.98	0.19	17.19	29.83	8.67	49.74	16.48
Mean		22.41	93.28	0.23	20.99	32.03	10.29	38.86	21.13
Budongquan To Naqu	14	27.19	104.47	0.42	37.87	27.77	9.38	26.03	18.07
	15	21.74	88.74	0.29	24.97	30.92	10.22	29.65	18.32
	16	25.58	150.78	0.86	73.55	36.40	11.01	31.26	32.39
	17	23.88	130.26	0.55	51.80	33.69	12.53	32.40	29.15
	18	16.39	82.72	0.32	27.00	31.76	8.40	30.31	18.97
Mean		22.95	111.39	0.49	43.04	32.11	10.31	29.93	23.38
Naqu To Lhasa	19	21.72	94.26	0.23	36.33	64.33	12.48	55.55	29.01
	20	18.68	106.80	0.19	34.68	39.22	8.46	30.23	11.22
	21	24.26	129.35	0.30	43.77	44.80	10.22	34.76	21.80
	22	24.35	105.26	0.24	33.13	41.76	11.09	33.51	24.62
	23	21.40	116.27	0.44	38.18	28.59	8.13	21.67	25.81
	24	20.83	98.42	0.21	39.46	31.12	8.81	24.35	21.24
Mean		21.87	108.39	0.27	37.59	41.64	9.87	33.35	22.28
Total		23.13	100.97	0.30	29.60	36.20	10.31	31.93	21.40

### 3.2. The Nonlinear Regression Models of Heavy Metals Concentrations

**Figure 2:**
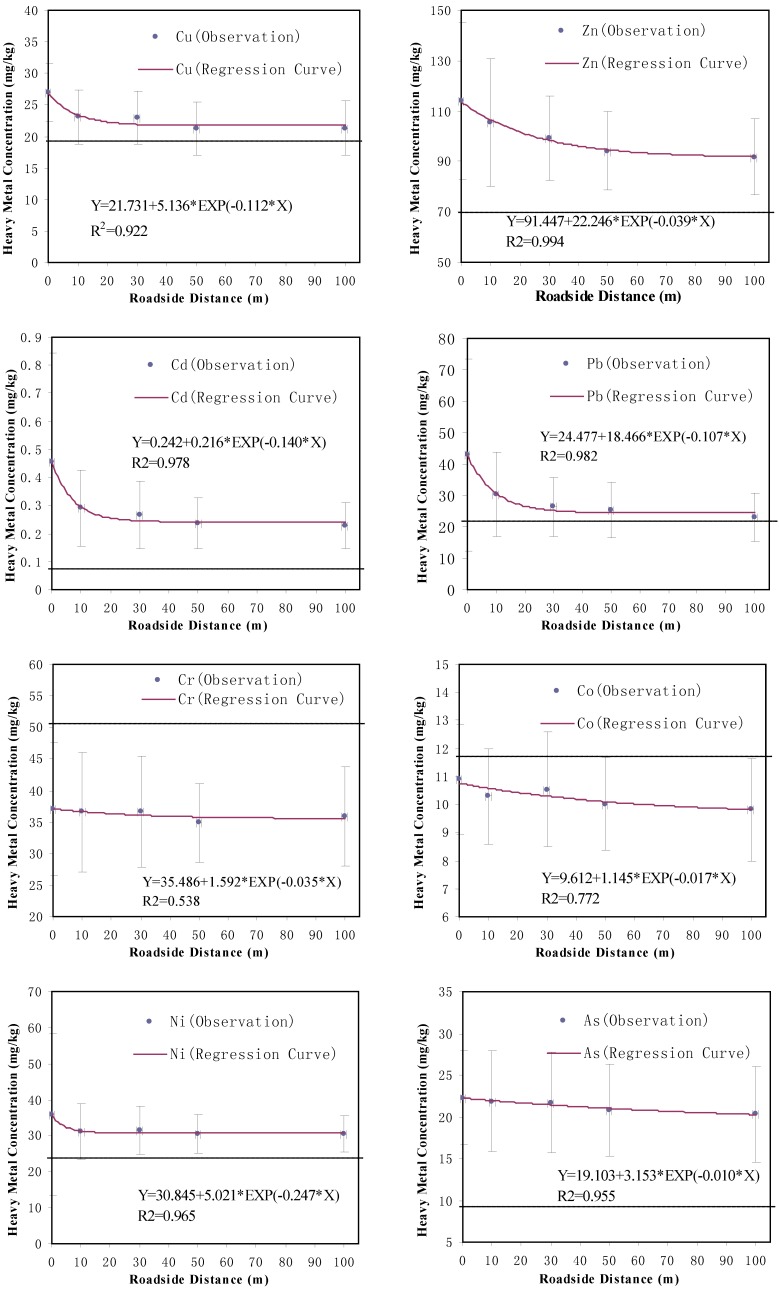
Observations and regression curves of heavy metals concentrations (mg·kg^−1^) in roadside soils (the dash lines represent background values).

The nonlinear regression method was applied to statistically characterize the relationship between heavy metal concentration and roadside distance. The regression curves of the metals’ concentrations based on roadside distance are shown in Figure 2. All of the concentrations of heavy metals investigated in this study decreased exponentially with the increase of distance to road edge. The predicted metal content is close to the observed values of heavy metals (relative errors are less than 10%). The R square values of the regression models are 0.922, 0.994, 0.978, 0.982, 0.538, 0.772, 0.965 and 0.955, respectively. They indicate that the exponential regression method can suitably describe the relationship between heavy metal concentration and roadside distance.

The concentration of Cu decreased totally by 5 mg·kg^−1^ to the relatively low value (22 mg·kg^−1^) at about 40 m from road edge, however, this was still higher than the background value (19.8 mg·kg^−1^). Cu mainly comes from brake wear and the mineral filler materials in asphalt road surfaces [26]. Most Cu particles are generated through the direct release of vehicle components rather than in exhaust gas, which deposit in the topsoil close to the road edge. 

The concentration of Zn gradually dropped to a relatively low value (91 mg·kg^−1^) at a distance of about 100 m from the road edge. Nabulo *et al.* [6] found that the concentration of Zn reached the background value at about 30 m from the road edge. However, in this study, the lowest Zn concentration was observed at the 100 m distance from the road edge, where the lowest concentration was still higher than the background value (91 mg·kg^−1^* vs*. 70 mg·kg^−1^). 

The concentration of Cd decreased with the increase of roadside distance and reaches a relatively low value (0.24 mg·kg^−1^) at 50 m from the road edge. In this study, the observed concentration values of Cd were much higher than the background value (0.084 mg·kg^−1^), even at 100 m from the road edge. 

Like Cd, the concentration of Pb decreased with the increment of roadside distance until a constant content of 24 mg kg^−1^ could be observed at 50 m from the road edge. This is the consistent with previous conclusions [27,28,29] that the contamination of Pb was mainly found within the range of 0 to 50 m away from roadside and then dropped to the background value at about 70 m or more roadside distance. 

The downward trend of Cr concentrations is not obvious. The regression curves of Co and As are very similar to those of Cr. The reduction percentage of the Co concentration from the road edge to 100 m away was around 10% and that of the As concentration was about 14%. However, the concentrations of Ni quickly reduced to a relatively low value (31 mg·kg^−1^) within a 10 m distance scope, which is consistent with the previous finding from Zehetner [2]. 

The comparison between previous conclusions and our present study are shown in Table 3. Nabulo *et al.* [6] found that the total trace metal (Pb, Cd and Zn) concentrations in roadside soils decreased exponentially as the roadside distance increased. Zehetner *et al.* [2] also reported that all metal contaminants (Pb, Cd, Cu, Zn, Ni and Cr) followed an exponential-like decrease with the increase of roadside distance, reaching background levels at 5 to 10 m distance from road edge. In our study, it was found that the maximum roadside distances that the contaminations can reach varied with different heavy metals. For example, the minimum concentrations of Zn and Cd at 100 m distance from road edge were still significantly higher than the background value, which indicates a larger roadside influential range than the previous studies. Therefore, a further roadside study is needed to investigate these metals’ distribution to identify how far these contaminants can actually reach. However, Zhang *et al.* [9] reported that the exponentially decreasing distribution pattern was not available in farm land soil due to agricultural activities and mixed metal resources. In the Qinghai-Tibet Plateau, the industrial facilities are very limited and its fossil fuel consumption is relatively low [30]. Especially, the sampling sites in this study were off residential, agricultural and industrial activities. Thus, the extra heavy metals accumulations in the roadside soils should directly result from traffic activities and the heavy metals’ distance-distribution patterns were rarely disturbed by the other potential pollution sources. 

**Table 3:** Previous studies of heavy-metal distribution patterns in roadside soils.

Location	Study area	Heavy metals	Distribution pattern with roadside distance	Reference
Benin, Newzealand	highway	Cu, Zn, Cd, Pb, Cr, Ni	decreased rapidly	[10]
Greater Athens Area	-	Zn, Cd, Pb, Ni	decreased exponentially and dropped to a background level at about 50 m	[18]
France	highway	Cu, Zn, Cd, Pb, Ni, Cr	decreased rapidly	[11]
Osogbo, Nigeria	-	Cu, Zn, Cd, Pb, Ni	decreased rapidly and reached the natural background levels at 50 m	[19]
Nancy, France	highway	Zn, Cd, Pb	decreased	[7]
Kampala, Uganda	highway	Zn, Cd, Pb	decreased rapidly	[6]
Northern England	A-and B-class roads	Cu, Zn, Cd, Pb	decreased	[8]
Beijing, China	10 main roads	Cu, Zn, Cd, Pb, Cr, Ni, As	decreased (except for As, Cr and Ni)	[3]
Turkey	highway	Cu, Pb, Cr	decreased (except for Cu)	[4]
Kathmadu, Nepal	highway	Cu, Zn, Cd, Pb	irregular	[9]
Qinghai-Tibet Plateau, China	highway	Cu, Zn, Cd, Pb, Cr, Co, Ni, As	decreased exponentially	**This study**

### 3.3. The Potential Ecological Risk Assessment of Heavy Metals in Roadside Soils

Metal contaminants in soils can have serious implications for the soil ecosystem, soil organisms and human health. It is necessary to evaluate the extent of the risks to potential receptors [31]. The Hakanson potential ecological risk index method can provide a quantitative evaluation on the potential ecological risk for a given contamination situation [24]. This method not only takes the pollution degrees of heavy metals into account, but also indicates the biological hazard degrees. Therefore, this evaluation method is a relatively stable, fast, simple and standard method [32]. Table 4 summarizes the potential ecological risk factors (Er^i^) and risk indices (RI) for each sampling site, each road segment, and the entire region, based on the mean metals concentrations in roadside soils. The values (Er^i^) of all metals except Cd were below 40, which indicatez that all investigated heavy metals except Cd posed no potential hazard to the local soil system. The Er^i^ distribution of Cd in the entire region was: 29% of sampling sites had a moderate potential ecological risk, 50% of sampling sites had a considerable potential ecological risk and 8% of the sites had a high potential ecological risk. On average, the rank of risk contribution rate (Er^i^/RI) among the heavy metals in a descending order is Cd > As > Ni > Pb > Cu > Co > Zn > Cr. Thus, Cd made the largest contribution to the total risk index while Cr made the smallest contribution. Since Cd is more highlighted than other elements in terms of the ecological risk factor in the region, it should be paid special attention to. 

**Table 4:** The potential ecological risk factors and risk indices of metals based on different road segments in roadside soils.

Road Segment	Site	Er^i^									
		Cu	Zn	Cd	Pb	Cr	Co	Ni	As	RI	Degree
Xining To Maduo (X-M)	1	7.16	1.44	123.29 **	5.49	1.42	4.24	5.72	26.72	175.48	moderate
	2	7.29	1.17	83.21 **	4.18	1.52	4.50	6.32	19.72	127.91	low
	3	6.32	1.23	92.43 **	4.61	1.42	4.29	5.96	18.47	134.72	low
	4	6.46	1.27	83.93 **	4.65	1.57	4.47	6.35	22.10	130.80	low
Mean		6.81	1.28	95.72 **	4.73	1.48	4.38	6.09	21.75	142.23	low
Maduo To Qumalai (M-Q)	5	5.16	1.24	72.00 *	4.26	1.29	4.26	6.15	20.65	115.01	low
	6	5.32	1.39	61.29 *	3.54	1.62	3.69	6.71	16.33	99.90	low
	7	5.90	1.33	98.29 **	4.68	1.45	4.45	6.51	26.22	148.83	low
	8	4.83	1.26	70.14 *	3.99	1.03	3.79	5.67	18.28	108.99	low
	9	6.67	1.67	81.29 **	5.61	1.49	6.16	8.07	31.53	142.48	low
	10	6.00	1.38	77.71 *	5.14	1.37	4.99	6.94	26.67	130.20	low
Mean		5.65	1.38	76.79 *	4.54	1.38	4.56	6.68	23.28	124.24	low
Qumalai To Budongquan (Q-B)	11	5.93	1.33	82.21 **	5.00	1.35	4.72	7.58	26.10	134.23	low
	12	6.65	1.52	96.36 **	5.23	1.24	4.69	6.76	27.22	149.67	low
	13	4.40	1.14	67.14 *	3.84	1.17	3.68	10.67	18.73	110.76	low
Mean		5.66	1.33	81.90 **	4.69	1.25	4.36	8.34	24.02	131.55	low
Budongquan To Naqu (B-N)	14	6.87	1.49	151.71 **	8.45	1.09	3.98	5.59	20.54	199.71	moderate
	15	5.49	1.27	103.64 **	5.57	1.21	4.33	6.36	20.82	148.70	low
	16	6.46	2.15	306.86 ****	16.42	1.42	4.67	6.71	36.81	381.50	considerable
	17	6.03	1.86	197.71 ***	11.56	1.32	5.31	6.95	33.12	263.87	moderate
	18	4.14	1.18	115.93 **	6.03	1.24	3.56	6.50	21.56	160.14	moderate
Mean		5.80	1.59	175.17 **	9.61	1.26	4.37	6.42	26.57	230.78	moderate
Naqu To Lhasa (N-L)	19	5.48	1.35	82.29 **	8.11	2.52	5.29	11.92	32.97	149.92	low
	20	4.72	1.53	67.14 *	7.74	1.53	3.59	6.49	12.75	105.48	low
	21	6.13	1.85	108.64 **	9.77	1.75	4.33	7.46	24.77	164.70	moderate
	22	6.15	1.50	84.07 **	7.39	1.63	4.70	7.19	27.98	140.63	low
	23	5.41	1.66	157.14 **	8.52	1.12	3.44	4.65	29.33	211.27	moderate
	24	5.26	1.41	74.79 *	8.81	1.22	3.73	5.23	24.14	124.58	low
Mean		5.53	1.55	95.68 **	8.39	1.63	4.18	7.16	25.32	149.43	low
Total		5.89	1.426	105.052	6.392	1.4	4.37	6.938	24.188	155.646	moderate

* Moderate risk; **considerable risk; *** high risk; **** very high risk.

The order of the mean potential risk indices (RI) for the five road segments is B-N > N-L > X-M > Q-B > M-Q. The risk level of B-N road segment was moderate, while the other four segments’ risk levels were low. The result can be explained by the fact that the traffic volume in the road segments from Budongquan to Lhasa was higher than the road segments from Xining to Budongquan, while the traffic volume in the M-Q segment is lowest. Intuitively, the heavy metal concentrations are significantly related to traffic volume [3]. 

**Figure 3:**
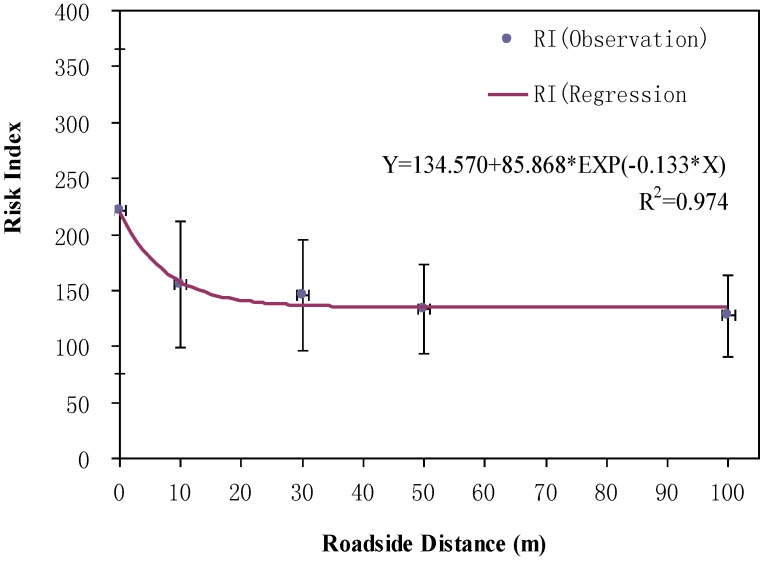
The mean risk indices based on different roadside distances of all sampling sites.

Very few studies have applied mathematical models to characterize the relationship between heavy metals’ potential ecological risk index and roadside distance. Such a modeling effort is meaningful for making related policies on how to avoid planting corps in roadside plots with a high pollution risk. In this study, the nonlinear exponential regression method was also applied to statistically model the spatial relationship. As shown in Figure 3, it presents an exponential decrease trend in the mean risk index with the increment of distance to road edge (R square is 0.974). The risk index had a clear decreasing trend from 220 to 135 within the roadside scope of 0-40 m distance. The moderate risk level (RI > 150) fell within the distance of 10 m. This is different from the conclusion in Li’s study [33], in which even at the road edge the risk was still in a low level (RI < 150) because the shelter forest along the roads could prevent the heavy metals’ depositions at a further distance. However, another study suggested that the safe roadside distance for farming should be 30 m [34] away from road edge based on the observational results. According to our study, a proper land use policy is required to inhibit the use of road border zone, especially within 10 m of distance to road edge, for cultivating, grazing or activities related to agriculture. Previous studies have reported that the safe roadside distance for farming or gazing is 30 m [34] base on heavy metal concentrations. 

## 4. Conclusions

Owing to the Chinese environmental protection policies and low population density in the Qinghai-Tibet Plateau, its environments are the least affected by anthropogenic interference. Therefore, the local contamination resulting from the increasing transportation activities in the region is receiving more attention. In this study, we investigated the contents of eight heavy metals (Cu, Zn, Cd, Pb, Cr, Co, Ni and As) in roadside soils along five major road segments across the Qinghai-Tibet Plateau. Because the sampling sites are off residential, agricultural and industrial activities, the extra heavy metals accumulations in roadside soils are almost purely due to traffic activities, which is helpful to assess the degree of traffic pollution and clarify the spatial relationships between the heavy metals’ accumulation and roadside distance.

According to the study results, the mean concentrations of Cu, Zn, Cd, Pb, Ni and As for the entire region were higher than its background values while the concentrations of Cr and Co were lower than the background values. Among the five studied road segments, the Budongquan-Naqu-Lhasa road segments constitute the most important transportation route in the Qinghai-Tibet Plateau, so it not surprising that the concentrations of Zn, Cd and As along the Budongquan-Naqu road segment and the concentrations of Pb and Cr along the Naqu-Lhasa road segment were found to be higher than the other road segments. The Hakanson potential ecological risk index calculation results showed that there was a moderate level of potential ecological risk within a 10 m roadside distance for the entire region. However, Cd was the only prominent heavy metal which contributed to the risk. The observed average concentration values of Cd were much higher than the background value of the region (0.30 mg·kg^−1^* vs.* 0.084 mg·kg^−1^), even at 100 m from the road edge (0.23 mg·kg^−1^* vs.* 0.084 mg·kg^−1^). Overall, the rank of risk contribution to the local environments among the eight heavy metals was Cd > As > Ni > Pb > Cu > Co > Zn > Cr. Considering Cd is a more hazardous heavy metal than of the other elements for public health, so the local government should pay special attention to this traffic-related environmental issue.

Especially, this study focused on modeling the relationships of heavy metals concentrations and potential ecological risk indices to the roadside distance. The regression results showed that both of the heavy metals’ concentrations and their ecological risk indices decreased exponentially with the increase of roadside distance, and the R square values for most of the exponential regression models were larger than 0.9, except for Cr and Co. Unfortunately, the specific daily traffic volume data for the sampling road segments were not available to this study. In the future, the more accurate models for predicting heavy metals concentrations in roadside soils and potential ecological risk should be developed based on both roadside distance and traffic volume of road segments. 
